# Supplementary material for: ABA-mediated regulation of rice grain quality and seed dormancy via the NF-YB1-SLRL2-bHLH144 Module
Source: Nat Commun. 2024 May 27;15:4493. doi: 10.1038/s41467-024-48760-w (PMC11130328; doi:10.1038/s41467-024-48760-w)
Supplement: Supplementary file 1 — Supplementary information [file 41467_2024_48760_MOESM1_ESM.pdf]

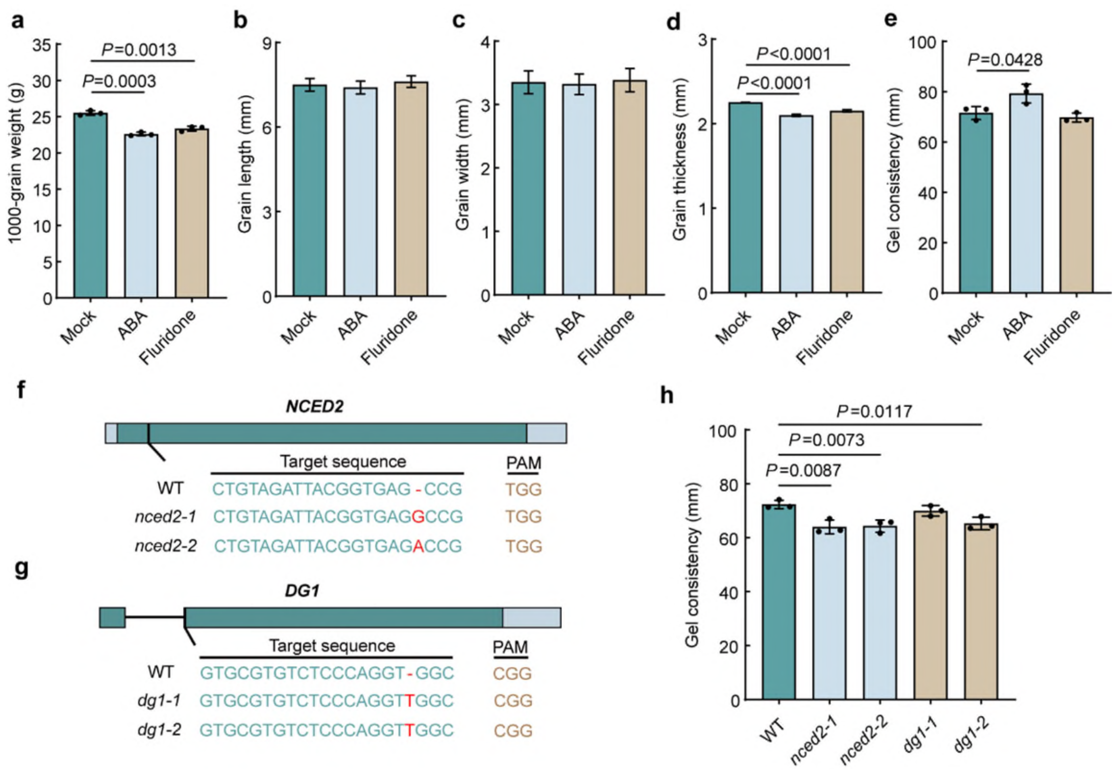

Supplementary Fig.1 Rice yield and quality traits in response to ABA or fluridone treatment. **a** 1000 grain weight, **b** grain length, **c** grain width, **d** grain thickness**, e** GC. Plants treated with alcohol mock served as controls. Data in **a-e** are means ± SD (n = 3 biological replicates in **a** and **e**; n= 20 biological replicates in **b**, **c** and **d**). Schematic diagram of the gene structure of *NCED2* **(f)** and *DG1* **(g)**, and their respective CRISPR/Cas9 gene editing target information. **h** GC of the *nced2* and *dg1* mutants. Statistical analysis was performed using two-tailed Student's *t*-test.

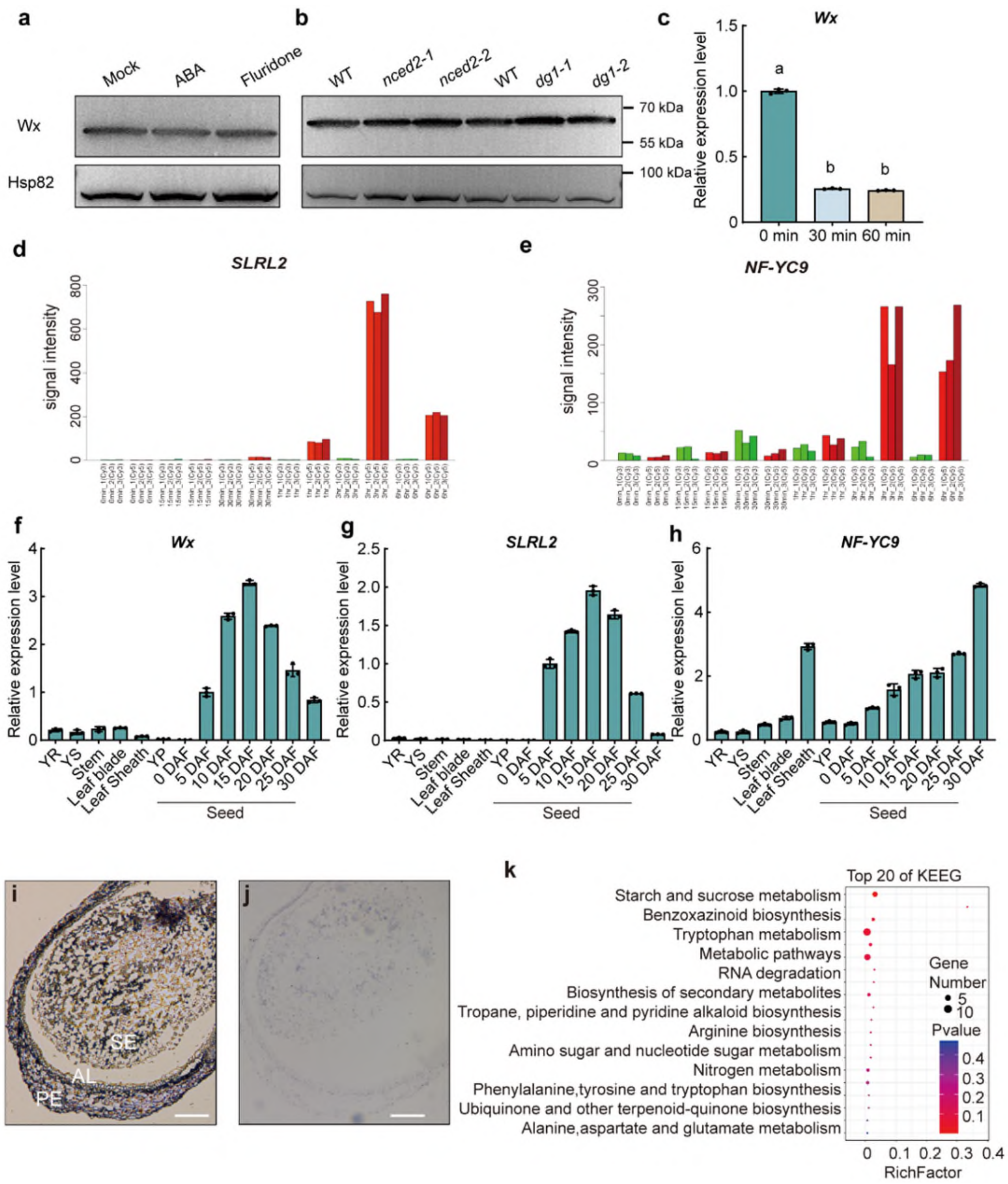

Supplementary Fig.2 Expression analysis of candidate genes mediating the regulation of *Wx* expression by ABA. a Wx protein levels after ABA or fluridone treatment. **b** Wx protein levels in the *nced2* and *dg1* mutants. The detection of Heat Shock Protein 82 (HSP82) was used as an internal control to normalize the protein load. The Wx and HSP82 proteins were detected by using anti-Wx and anti-HSP82 antibodies, respectively. **c** *Wx* expression under short-term ABA treatment. The 14-d-old rice seedlings were transferred to a medium containing 50 μM ABA, and rice samples were collected at 0, 30 and 60 min after ABA treatment, respectively. *OsUBC* was used as an internal control to normalize gene expression. The expression level of *Wx* was set to 1 for samples without ABA treatment. Data are means ± SD (n = 3

biological replicates). Different letters denote significant differences ($P < 0.05$, one-way ANOVA with two-sided Tukey's HSD test). *P* values are adjusted and shown in the Source Data file. Expression analysis of *SLRL2* **(d)** and *NF-YC9* **(e)** in response to ABA treatment. Spatio-temporal expression patterns of *Wx* **(f)**, *SLRL2* **(g),** and *NF-YC9* **(h)** in different tissues of rice. *OsActin01* was used as an internal control to normalize gene expression. The expression level of each gene in seeds at 5 days after flowering (DAF) was set to 1. Young root (YR) and young shoot (YS) samples were collected from 14-day-old rice seedlings. Stem, leaf blade, leaf sheath, and young panicle (YP) samples were all from rice at the booting stage. Developing seed samples were collected at 0, 5, 10, 15, 20, 25, and 30 DAF. Data in **f-h** are means ± SD (n = 3 biological replicates). **i** In situ hybridization analysis of *SLRL2* expression in rice seeds at 5 DAF. SE, starchy endosperm; AL, aleurone layer; PE, pericarp. The scale bar is 500 μm. **j** Negative control for *SLRL2* in situ hybridization. **k** KEEG enrichment analysis.

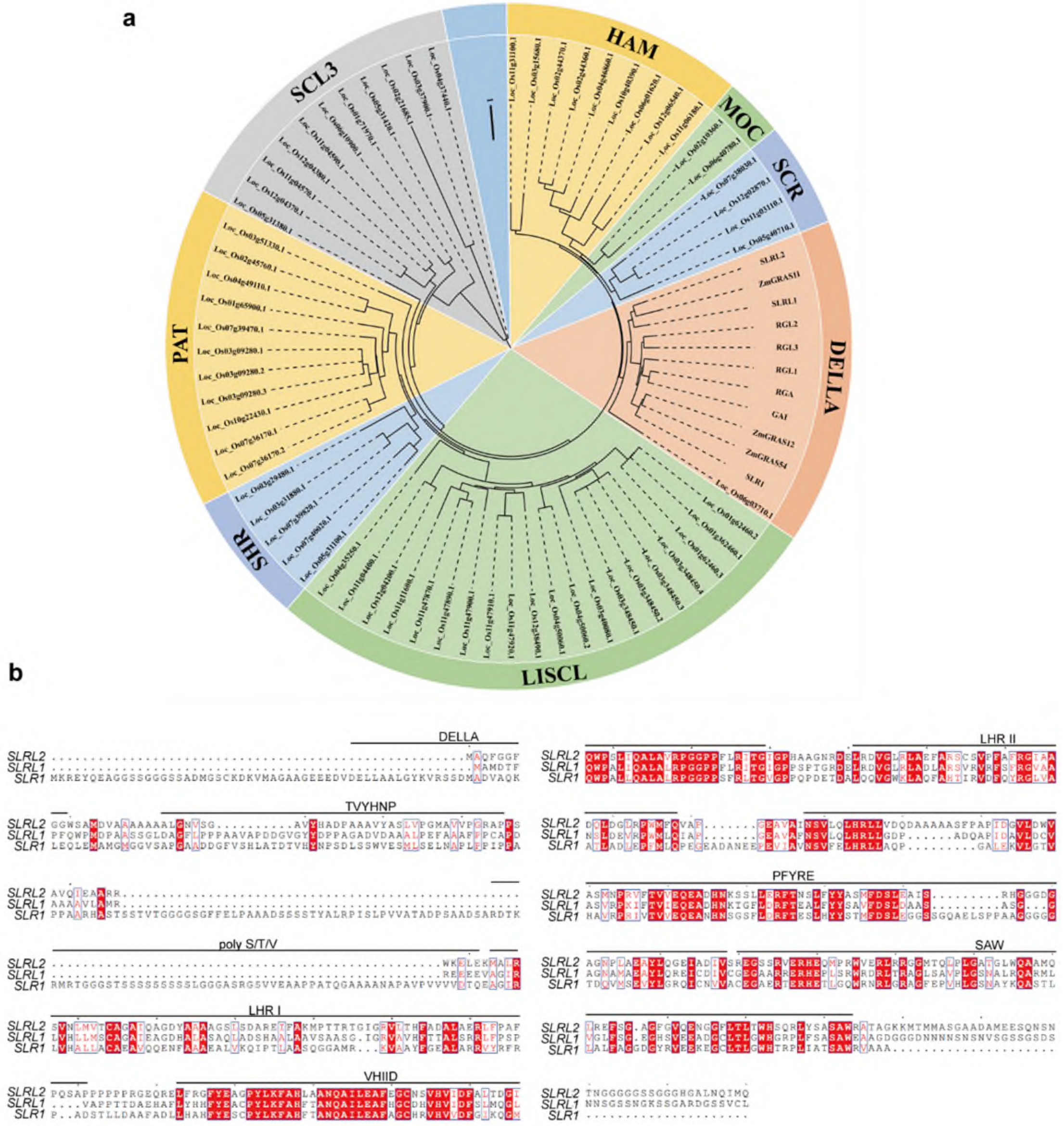

Supplementary Fig.3 Phylogenetic analysis of GRAS proteins in rice and DELLA family proteins from Arabidopsis and maize (a), and Clustal alignment of DELLA family members in rice (b).

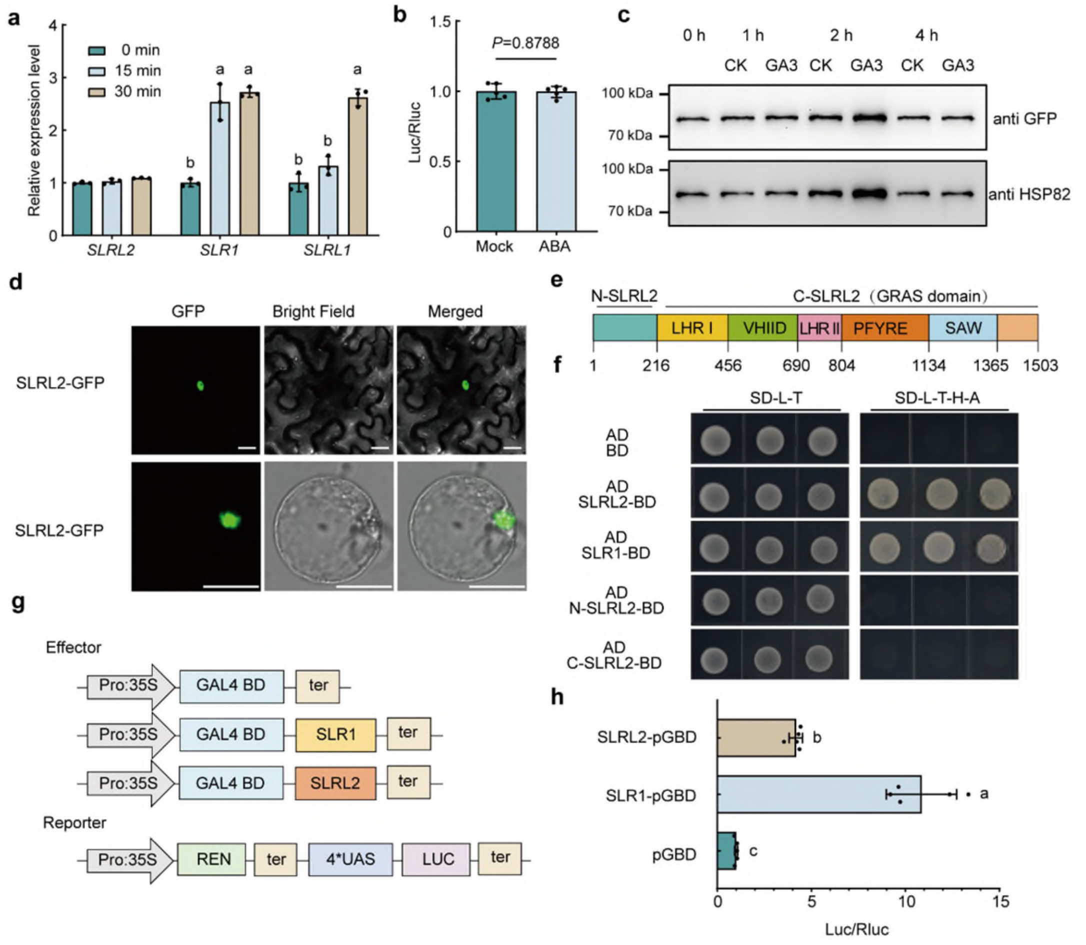

Supplementary Fig.4 Expression analysis of *SLRL2*, and analysis of subcellular localization and transcriptional activity of SLRL2. a RT-qPCR analysis of the transcription of *SLR1, SLRL1,* and *SLRL2* in response to GA treatment. The 14-day-old rice seedlings were treated with 50 µM GA. Rice seedling samples for expression assay were collected at the indicated time point. *OsUBC* served as an internal control for the normalization of gene expression data. The transcript abundance of each gene in the samples before GA treatment was set to 1. Data are means ± SD (n = 3 biological replicates). Different letters denote significant differences ($P < 0.05$, one-way ANOVA with two-sided Tukey's HSD test). *P* values are adjusted and shown in the Source Data file. **b** Dual luciferase reporter assay was used to investigate the response of *SLRL2* to GA treatment. The fluorescence ratio was measured after treatment with 50 µM GA for

30 min. Data are means ± SD (n = 5 biological replicates) and comparisons are made by two-tailed Student's *t*-test. **c** The abundance of SLRL2 protein in response to GA treatment. Hsp82 was used as an internal control to normalize the protein loading. The SLRL2-GFP and HSP82 proteins were detected by using anti-GFP and anti-HSP82 antibodies, respectively. **d** Analysis of SLRL2 subcellular localization in both tobacco epidermal cells and rice protoplasts. The scale bar is 10 μM. **e** Schematic representation of the conserved domain of the SLRL2 protein. **f** Analysis of the transcriptional activities of SLR1, SLRL2, N-SLRL2, and C-SLRL2 in yeast cells. Constructs (**g)** and result (**h)** for transcriptional activity analysis of SLRL2 and SLR1 by using the dual luciferase system in rice protoplasts. Data are means ± SD (n = 5 biological replicates). Different lowercase letters above error bars indicate significant differences as determined by one-way ANOVA with Tukey's HSD test. *P* values are shown in the Source Data file.

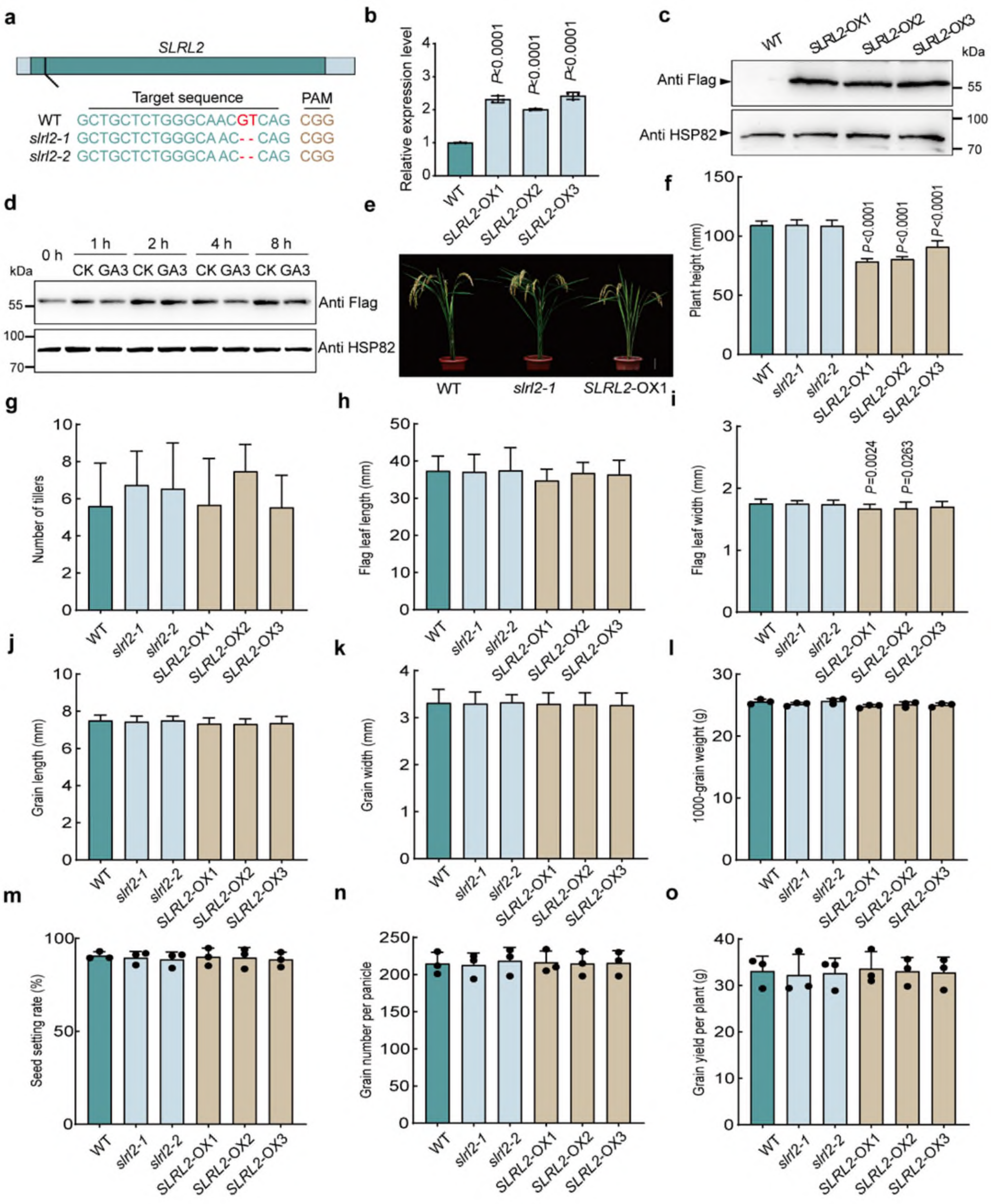

Supplementary Fig.5 Agronomic trait analysis of *slrl2* mutant and *SLRL2* overexpression transgenic rice. a Schematic diagram of the *SLRL2* gene structure and the corresponding CRISPR/Cas9 gene editing target information. Light blue color represents untranslated regions and green color represents coding regions. **b** RT-qPCR analysis of *SLRL2* in *SLRL2*-overexpressing transgenic rice and its WT control. *OsActin01* was used as an internal control to normalize the gene expression. *SLRL2* transcript abundance in the WT control was set to 1. Data are means ± SD (n = 3 biological replicates). **c** Western blot analysis of SLRL2 protein in transgenic rice overexpressing *SLRL2*. Detection of HSP82 was used as an internal control for

normalization of protein loading. **d** Analysis of SLRL2 protein in response to GA treatment. The seedlings of transgenic rice overexpressing *SLRL2* were used for GA treatment and subsequent Western blot analysis. Hsp82 was used as an internal control to normalize the protein loading. SLRL2-Flag and HSP82 proteins were detected by using anti-Flag and anti-HSP82 antibodies, respectively. **e** Phenotypes of representative *SLRL2* knockout and overexpression transgenic plants and the WT control. The scale bar is 10 cm. **f** Quantitative data of rice plant height. Tiller number (**g**), flag leaf length (**h**), flag leaf width (**i**), grain length (**j**), grain width (**k**), 1000 grain weight (**l**), seed setting rate (**m**), grain number per panicle (**n**), and grain yield per plant (**o**) of *SLRL2*-related rice materials. Data in **f-o** are means ± SD (n = 15 biological replicates in **f-k**; n=3 biological replicates in **l-o**.). Statistical analysis was performed by two-tailed Student's *t*-test.

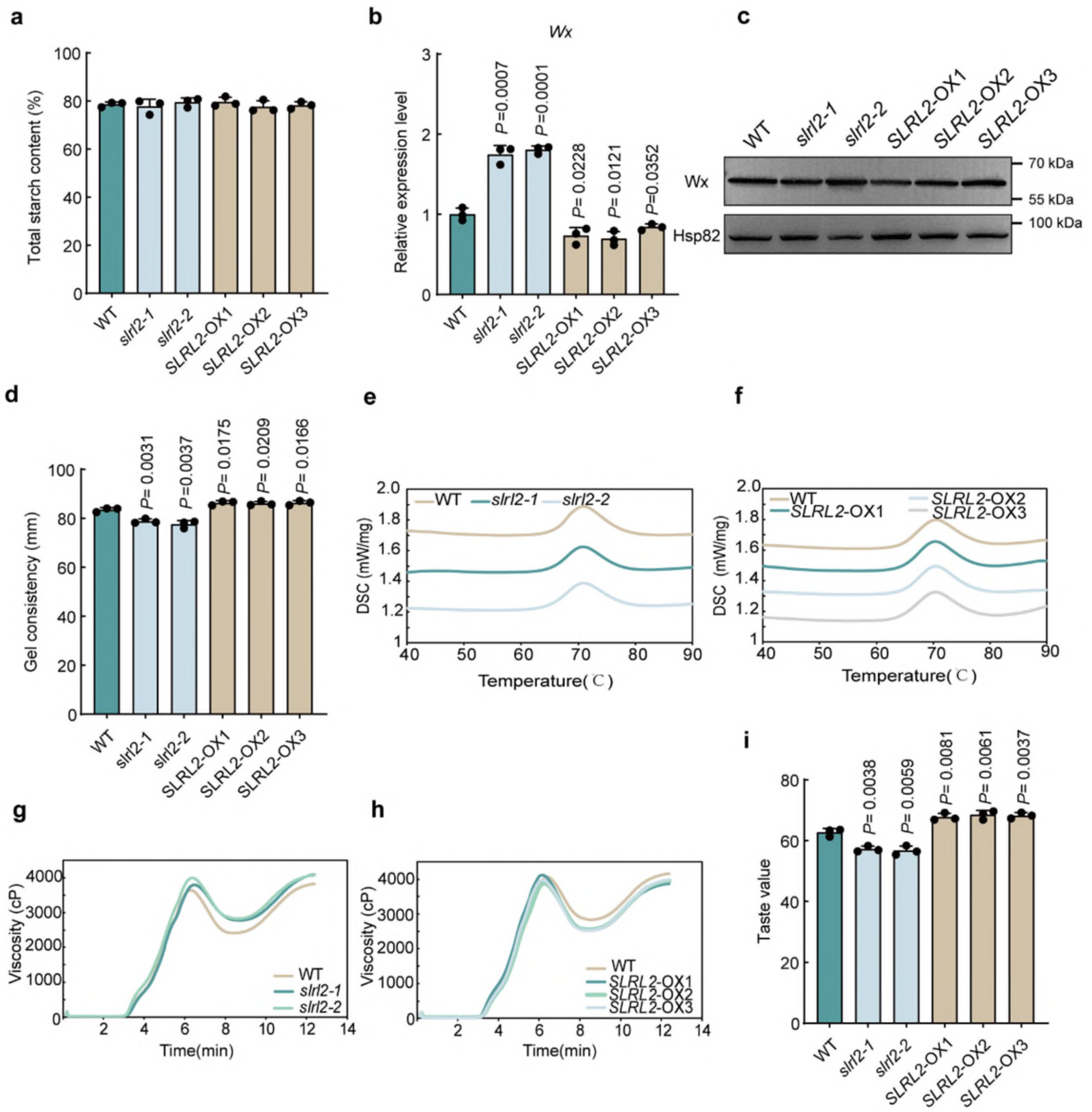

Supplementary Fig.6 Analysis of *Wx* gene expression and starch physicochemical properties and palatability of *SLRL2*-related rice materials. a Total starch content of the *SLRL2* knockout and overexpression transgenic plants and the WT control. Data are means ± SD (n = 3 biological replicates)**. b** *Wx* gene expression levels in *SLRL2*-related materials. *OsActin01* was used as an internal control to normalize gene expression. The transcript abundance of *Wx* in the WT was set to 1. Data are means ± SD (n = 3 biological replicates). **c** Analysis of Wx protein abundance in *SLRL2*-related rice materials. **d** GC of the *SLRL2* knockout and overexpression transgenic plants. Data are means ± SD (n = 3 biological replicates). Differential scanning calorimetry (DSC) analysis of *slrl2* mutant (**e)** and *SLRL2* overexpression transgenic rice (**f**). Rapid viscosity analyzer (RVA) analysis of *slrl2* mutants (**g)** and *SLRL2* overexpression

transgenic rice (**h**). **i** Taste scores of the *SLRL2* knockout and overexpression transgenic plants and the WT control. In **d** and **I**, data are means ± SD biologically independent experiments. A two-sided Student's paired t-test was used to generate the *P*-values in **a, b, d** and **i.**

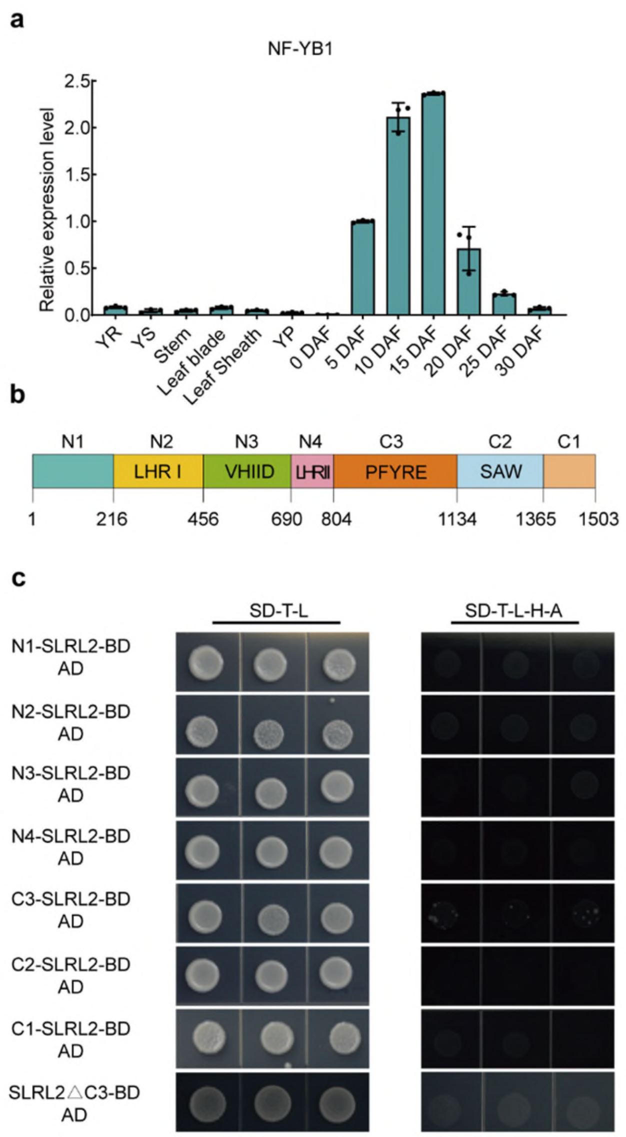

Supplementary Fig.7 Spatiotemporal expression pattern of *NF-YB1* gene in rice, and the transcriptional activity of each domain of SLRL2. a *OsActin01* was used as an internal control for normalization of gene expression data. The expression level of *NF-YB1* in seeds five days after flowering (DAF) was set to 1. Young root (YR) and young shoot (YS) samples were collected from 14-day-old rice seedlings. Stem, leaf blade, leaf sheath, and young panicle (YP) samples were all from rice at the booting stage. Developing seed samples were collected at 0, 5, 10, 15, 20, 25, and 30 DAF. Data are means ± SD (n = 3 biological replicates). **b** Schematic representation of SLRL2 protein and its different truncation forms for yeast two-hybrid assay. **c** Yeast two-hybrid analysis to examine the transcriptional activity of each truncation form of SLRL2 protein.

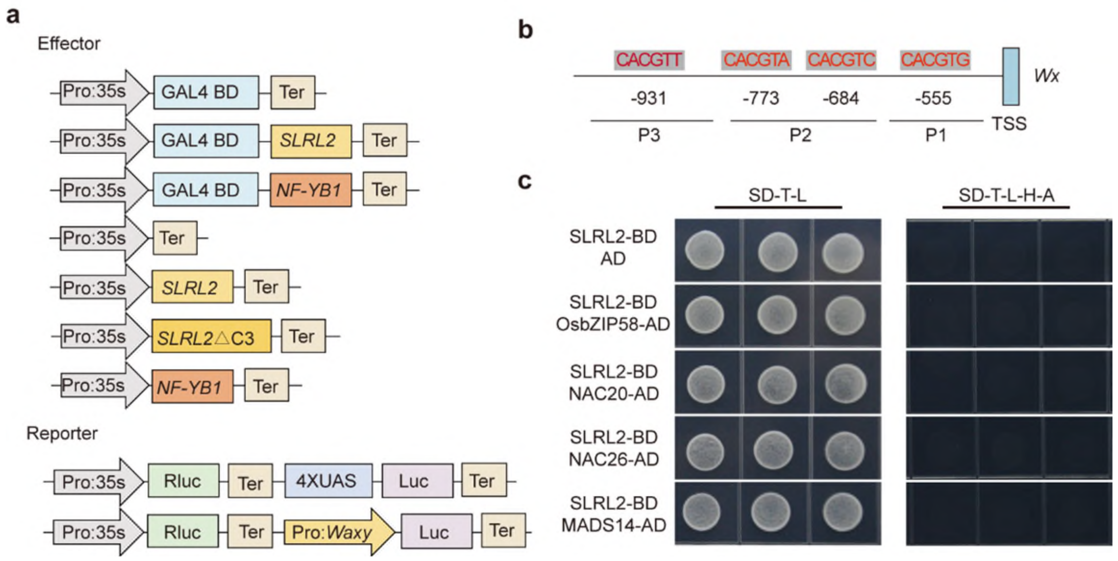

Supplementary Fig.8 Schematic diagrams of the vectors required for dual-luciferase reporter gene experiments and the *Wx* gene promoter, and investigation of the potential interaction between SLRL2 and some other Wx-regulation-related transcription factors. a Schematic diagram of the vectors required for dual-luciferase reporter experiments with SLRL2 and NF-YB1. **b** Schematic representation of the *Wx* promoter highlighting the G-box motif regions. The marked regions P1 to P3 were the positions used for the ChIP-qPCR assay. **c** Investigation of the potential interaction between SLRL2 and some other *Wx* regulation-related transcription factors, including bZIP58, NAC20, NAC26, and MADS14.

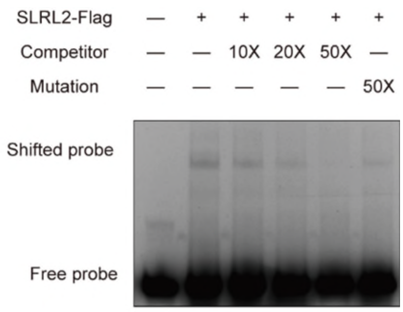

Supplementary Fig.9 EMSA experiments showed the direct binding of SLRL2 to the *Wx* promoter.

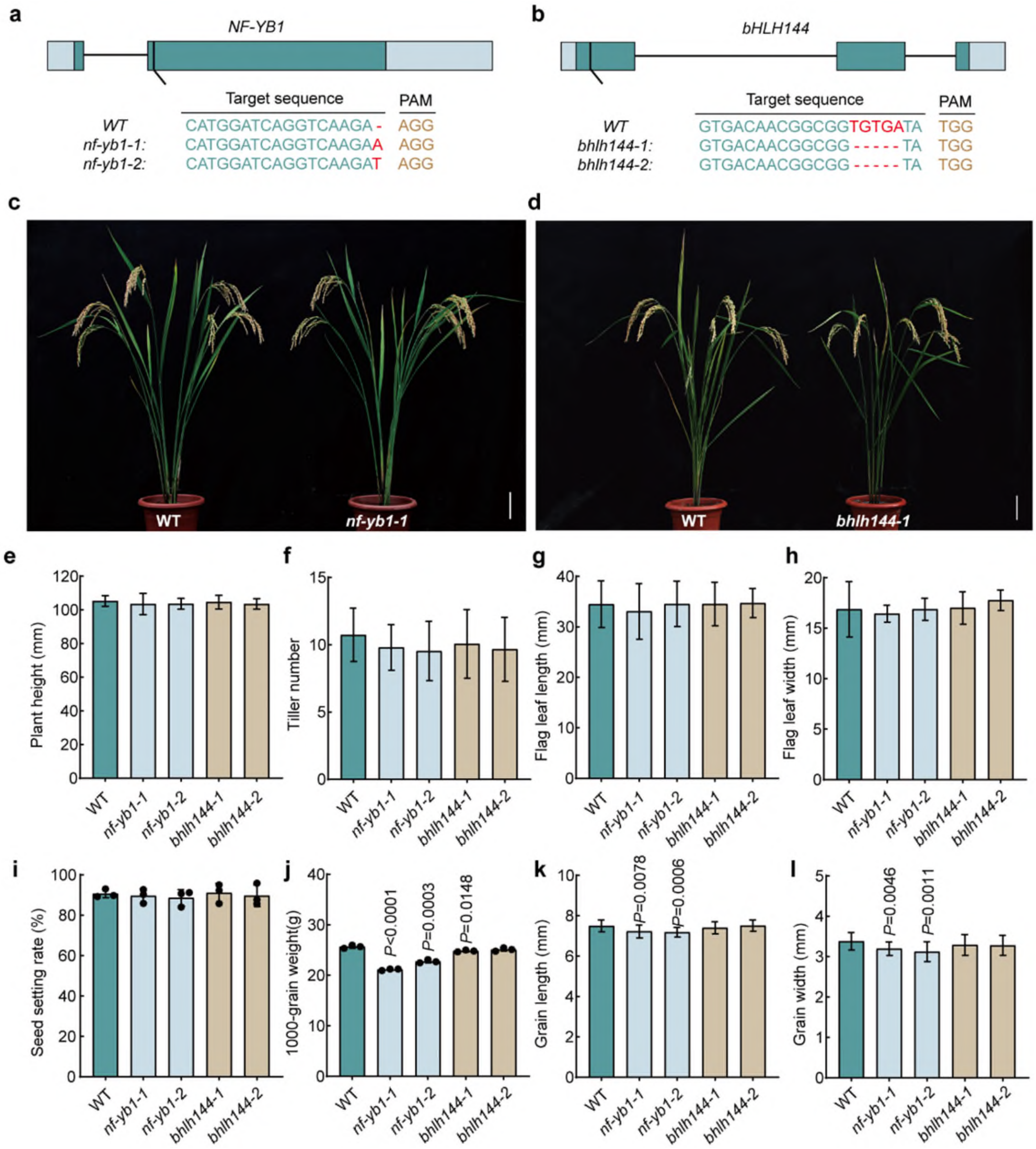

Supplementary Fig.10 Generation and agronomic trait analysis of *nf-yb1* and *bhlh144* rice mutants. Schematic diagram of the gene structure of *NF-YB1* (**a**) and *bHLH144* (**b**) and their corresponding CRISPR/Cas9 gene editing target information. Light blue color represents untranslated regions and green color represents gene coding regions. Phenotypes of rice mutants *nf-yb1* (**c**) and *bhlh144* (**d**). The scale bar is 10 cm. The plant height (**e**), tiller number (**f**), flag leaf length (**g**), flag leaf width (**h**), seed setting rate (**i**), 1000 grain weight (**j**), grain length (**k**), and grain width (**l**) of rice mutant *nf-yb1* and *bhlh144*. Data in **e-l** are means ± SD (n = 15 biological replicates in **e**, **f**, **g**, **h**, **k** and **l**; n= 3 biological replicates **i** and **j**). A two-sided Student's paired t-test was used to generate the *P*-values.

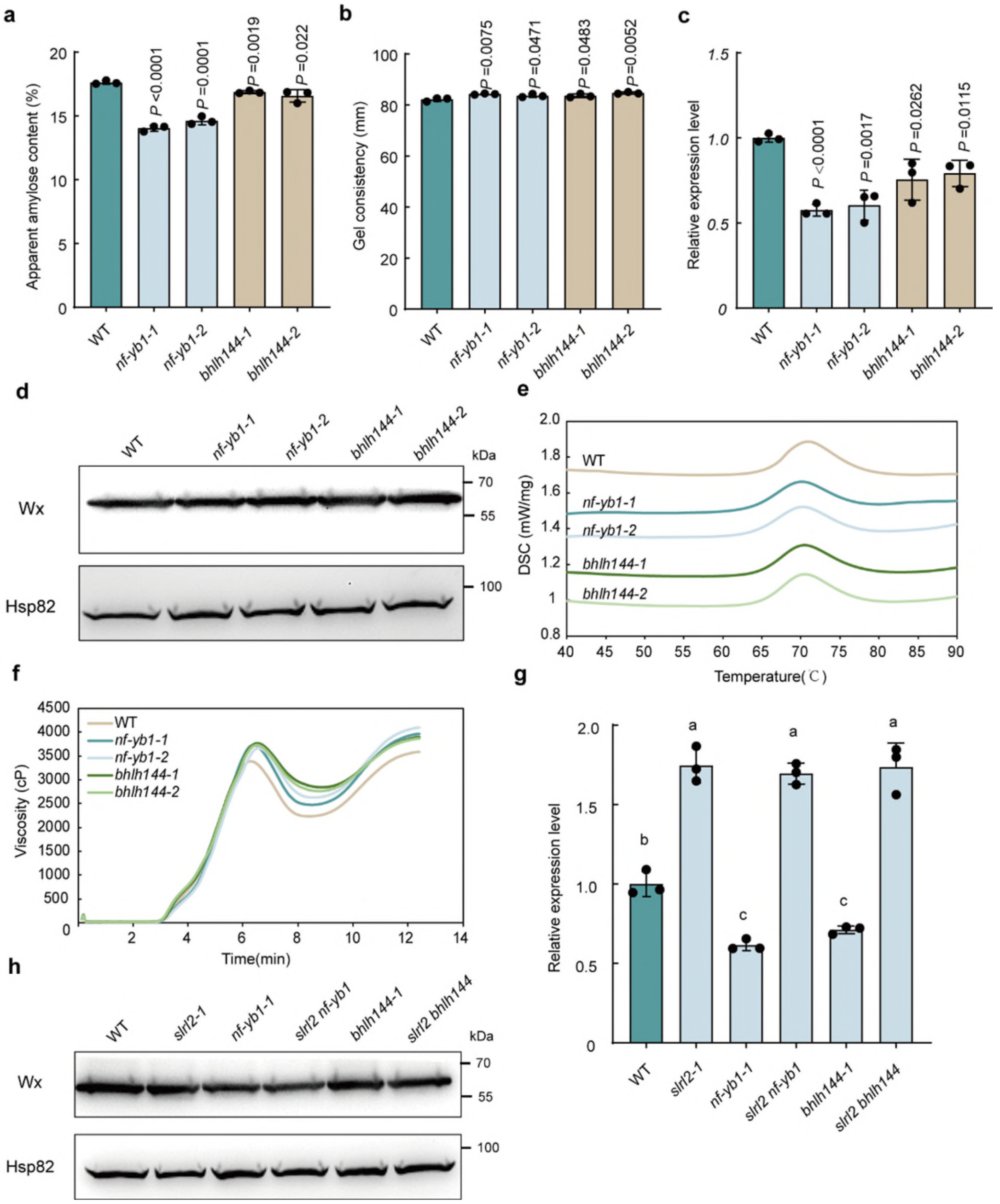

Supplementary Fig.11 Wx expression analysis and starch physicochemical properties of *NF-YB1*, *bHLH144,* and *SLRL2*-related mutants. AC (**a**) and GC (**b**) of *nf-yb1* and *bhlh144* rice mutants. **c** Transcriptional analysis of the *Wx* gene in *nf-yb1* and *bhlh144* mutants. Data in **a-c** are means ± SD (n = 3 biological replicates). A two-sided Student's paired t-test was used to generate the *P*-values in **a**, **b** and **c**. **d** Western blot analysis of Wx protein abundance in *nf-yb1* and *bhlh144* mutants. Gelatinization characteristics (**e**) and pasting properties (**f**) of rice from *nf-yb1* and *bhlh144* rice

mutants. **g** Transcriptional analysis of the *Wx* gene in the single and double rice mutants with respect to *SLRL2*, *NF-YB1*, and *bHLH144* genes. The expression of *Wx* gene in WT was set to 1. Data are means ± SD (n=3 biological replicates). A one-way ANOVA with two-sided Tukey's HSD test was used to generate the *P*-values. **h** Western blot analysis of Wx protein in the single and double rice mutants concerning *SLRL2, NF-YB1*, and *bHLH144* genes.

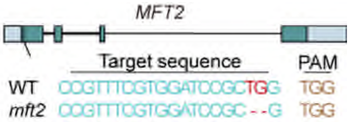

Supplementary Fig.12 Schematic diagram of the *MFT2* gene structure and corresponding CRISPR/Cas9 gene editing target information.
