# Supplementary material for: ABA-mediated regulation of rice grain quality and seed dormancy via the NF-YB1-SLRL2-bHLH144 Module
Source: Nat Commun. 2024 May 27;15:4493. doi: 10.1038/s41467-024-48760-w (PMC11130328; doi:10.1038/s41467-024-48760-w)
Supplement: Supplementary file 3 — Reporting Summary [file 41467_2024_48760_MOESM3_ESM.pdf]

nature portfolio

Corresponding author(s): Qian-Feng Li, Qiao-Quan Liu

Last updated by author(s): May 8, 2024

Reporting Summary

Nature Portfolio wishes to improve the reproducibility of the work that we publish. This form provides structure for consistency and transparency in reporting. For further information on Nature Portfolio policies, see our Editorial Policies and the Editorial Policy Checklist.

Statistics

For all statistical analyses, confirm that the following items are present in the figure legend, table legend, main text, or Methods section.

n/a	Confirmed	
☐	☒	The exact sample size (*n*) for each experimental group/condition, given as a discrete number and unit of measurement
☐	☒	A statement on whether measurements were taken from distinct samples or whether the same sample was measured repeatedly
☐	☒	The statistical test(s) used AND whether they are one- or two-sided<br>*Only common tests should be described solely by name; describe more complex techniques in the Methods section.*
☒	☐	A description of all covariates tested
☐	☒	A description of any assumptions or corrections, such as tests of normality and adjustment for multiple comparisons
☐	☒	A full description of the statistical parameters including central tendency (e.g. means) or other basic estimates (e.g. regression coefficient) AND variation (e.g. standard deviation) or associated estimates of uncertainty (e.g. confidence intervals)
☐	☒	For null hypothesis testing, the test statistic (e.g. *F*, *t*, *r*) with confidence intervals, effect sizes, degrees of freedom and *P* value noted<br>*Give P values as exact values whenever suitable.*
☒	☐	For Bayesian analysis, information on the choice of priors and Markov chain Monte Carlo settings
☒	☐	For hierarchical and complex designs, identification of the appropriate level for tests and full reporting of outcomes
☒	☐	Estimates of effect sizes (e.g. Cohen's *d*, Pearson's *r*), indicating how they were calculated

Our web collection on statistics for biologists contains articles on many of the points above.

Software and code

Policy information about availability of computer code

Data collection	The relative luciferase activity was measured using the dual luciferase assay kit (Vazyme, Jiangsu, China) and the TECAN Infinite M200 microplate reader. qRT-PCR was performed on the CFX Connect Real-Time PCR Detection System (Bio-Rad, California, USA) using the AceQ qPCR SYBR Green Master Mix (Vazyme, Jiangsu, China). The fluorescence signals were observed under a confocal laser scanning microscope (LSM 710; Carl Zeiss AG, Germany). The pasting properties of starch were determined using a differential scanning calorimeter (DSC 200 F3, Netzsch Instruments NA LLC; Burlington, MA). The pasting properties of rice were evaluated using a rapid viscosity analyzer (RVA) (Techmaster, Newport Scientific, Warriewood, Australia).
Data analysis	Phylogenetic analysis was performed using online software (https://www.omicshare.com/tools/Home/Soft/aa2tree). Protein homology alignment was performed using the online software ESPript 3.0 (https://espript.ibcp.fr/ESPript/cgi-bin/ESPript.cgi). KEGG enrichment of SLRL2 co-expressed genes was analyzed using online software (https://www.omicshare.com/tools/Home/Soft/pathwaygseasenior).

For manuscripts utilizing custom algorithms or software that are central to the research but not yet described in published literature, software must be made available to editors and reviewers. We strongly encourage code deposition in a community repository (e.g. GitHub). See the Nature Portfolio guidelines for submitting code & software for further information.

Data

Policy information about availability of data

All manuscripts must include a data availability statement. This statement should provide the following information, where applicable:

- Accession codes, unique identifiers, or web links for publicly available datasets
- A description of any restrictions on data availability
- For clinical datasets or third party data, please ensure that the statement adheres to our policy

All the relevant data supporting the findings of this work are available in this article and its Supplementary information files. All constructs and transgenic plants are available upon request. The gene sequences are available from RAPDB (https://rapdb.dna.affrc.go.jp/) using the accession numbers provided in this article. Source data are provided with this paper.

Research involving human participants, their data, or biological material

Policy information about studies with human participants or human data. See also policy information about sex, gender (identity/presentation), and sexual orientation and race, ethnicity and racism.

Reporting on sex and gender	None.
Reporting on race, ethnicity, or other socially relevant groupings	None.
Population characteristics	None.
Recruitment	None.
Ethics oversight	None.

Note that full information on the approval of the study protocol must also be provided in the manuscript.

Field-specific reporting

Please select the one below that is the best fit for your research. If you are not sure, read the appropriate sections before making your selection.

☒ Life sciences ☐ Behavioural & social sciences ☐ Ecological, evolutionary & environmental sciences

For a reference copy of the document with all sections, see nature.com/documents/nr-reporting-summary-flat.pdf

Life sciences study design

All studies must disclose on these points even when the disclosure is negative.

Sample size	No statistic methods were used to predetermine sample size. The required experimental sample size was estimated based on our past experience in conducting similar experiments, including field test (Huang et al.,Plant Biotechnol J.2020, 18: 2164-2166; Xiong et al.,Plant Physiol.2022, 189:402–418).
Data exclusions	No data were excluded from our analysis.
Replication	Five biological replicates were used for the dual luciferase system and PHS assay experiments. All other experiments were successfully replicated at least three times.
Randomization	Measurements and samplings were performed by randomly selecting plants grown under the exact same conditions.All samples were allocated randomly intoexperimental groups.
Blinding	We did not apply blinding as it was not applicable for the nature of the experimental setup.

Reporting for specific materials, systems and methods

We require information from authors about some types of materials, experimental systems and methods used in many studies. Here, indicate whether each material, system or method listed is relevant to your study. If you are not sure if a list item applies to your research, read the appropriate section before selecting a response.

Materials & experimental systems

n/a	Involved in the study
☐	☒ Antibodies
☒	☐ Eukaryotic cell lines
☒	☐ Palaeontology and archaeology
☒	☐ Animals and other organisms
☒	☐ Clinical data
☒	☐ Dual use research of concern
☐	☒ Plants

Methods

n/a	Involved in the study
☒	☐ ChIP-seq
☒	☐ Flow cytometry
☒	☐ MRI-based neuroimaging

Antibodies

Antibodies used

Anti-Flag monoclonal antibody (Sigma-Aldrich,Cat.#F3165,dilution 1:8000
Anti-Hsp82 monoclonal antibody(Beijin Protein Innovation(BPI),Cat.#AbM51099-31-PU,dilution 1:5000)
Anti-Myc antibody (Trans,Cat.#HT101,dilution 1:3000)
Anti-GFP Mouse Monoclonal Antibody(Trans,Cat.#HT801,dilution 1:3000)
Anti-OsGBSSI Rabbit Monoclonal Antibody(ABclonal,Cat.#A19151,dilution 1:3000).
Goat Anti-Mouse IgG-HPR secondary antibody CWBIO,Cat.#CW0102S,dilution 1:10000

Validation

The Anti-Flag antibody validation could be found in the website:https://www.sigmaaldrich.cn/CN/zh/product/sigma/f3165.
The Anti-Hsp82 monoclonal antibody validation could be found in the website:http://www.proteomics.org.cn/product/202.html.
The Anti-Myc antibody validation could be found in the website:https://www.transgen.com/antibody_tag/363.html.
The Anti-GFP Mouse Monoclonal Antibody validation could be found in the website:https://www.transgen.com/antibody_tag/390.html.
The Anti-OsGBSSI Rabbit Monoclonal Antibody validation could be found in the website: https://abclonal.com.cn/catalog/A19151.
The Goat Anti-Mouse IgG-HPR secondary antibody validation could be found in the website:https://www.cwbio.com/goods/index/id/10118
The Goat Anti-Rabbit IgG-HPR secondary antibody validation could be found in the website: https://www.solarbio.com/goodsInfo?id=1546

Plants

Seed stocks

The japonica rice cultivar ZH11 and NIP are widely used for transformation. The seeds of ZH11 and NIP were stored in our lab (Yangzhou University, Yangzhou, China).

Novel plant genotypes

o generate rice mutants using the CRISPR/Cas9 gene editing method, specific target sites for the genes SLRL2, NF-YB1, and bHLH144 were carefully designed and cloned into the pC1300-Cas9 vector, respectively. The endogenous sequences of SLRL2, NF-YB1, bHLH144 and MFT2 targeted for editing are as follows: GCTGCTCTGGGCAACGTCAG (SLRL2), CATGGATCAGGTCAAGAAGG (NF-YB1), GTGACAACGGCGGTGTGATA (bHLH144) and CCGTTTCGTGGATCCGCTGG (MFT2). The SLRL2 overexpression vector was constructed by cloning the SLRL2 coding sequence (CDS) into the binary vector pCAMBIA1300, driven by the OsActin01 promoter and fused to a flag epitope. The verified constructs were transformed into the recipient rice variety ZH11 by Agrobacterium tumefaciens-mediated transformation. For the rice mutants slrl2, nf-yb1, and bhlh144, two independent homozygous frameshift mutants of each gene were identified for subsequent analysis. Three independent representative homozygous lines of SLRL2-overexpressing rice, designated SLRL2-OX1, SLRL2-OX2, and SLRL2-OX3, were selected for the subsequent analyses.

Authentication

The rice mutants generated by gene editing were sequenced to detect the mutation types. The SLRL2-overexpressing rice plants were verified by genotyping and Western analysis.
